# Designing Soft and Transparent Films Based on Multi-Phase Polypropylene Copolymers and Styrene Block Copolymers

**DOI:** 10.3390/polym18091140

**Published:** 2026-05-06

**Authors:** Markus Gahleitner, Dietrich Gloger, Katja Klimke, Martina Sandholzer, Jingbo Wang

**Affiliations:** Borealis Polyolefine GmbH, St. Peterstr. 25, 4021 Linz, Austria; dietrich.gloger@borealisgroup.com (D.G.); sandholzer@raumbewegt.at (M.S.); jingbo.wang@borealisgroup.com (J.W.)

**Keywords:** polypropylene, film, medical applications, styrene elastomers, morphology, mechanics, optics

## Abstract

Concerns about the environmental and health impacts of plasticized poly (vinyl chloride) (PVC), from plasticizer loss to microplastic formation, have created a clear demand to find alternative packaging materials for medical and pharmaceutical use. As a possible polyolefin-based alternative, we blended polypropylene–ethylene copolymers with different ethylene content-controlled phase structures with styrene–ethylene/butylene–styrene block copolymer (SEBS), as modifier. SEBS is elastomeric and performs mechanically like a cross-linked rubber due to its unique microphase-separated morphology of hard spherical polystyrene (PS) domains dispersed in the soft elastomeric ethylene-butylene copolymer (EB) phase. Tests with injection-molded samples and cast films demonstrated promising combinations of flexibility, durability, and transparency—qualities essential for soft medical packaging like infusion pouches and blow–fill–seal bottles. For the desired level of flexibility (reflected by a flexural modulus of 150–250 MPa), blends with two random-heterophasic (RAHECO) copolymers achieved the lower limit with only 15–25 wt.-% SEBS, compared to the 37 wt.-% needed for a single-phase random copolymer (RACO). These blends also exhibited greater toughness and excellent transparency. In contrast, a standard impact copolymer (HECO), with its more crystalline matrix, required a higher modifier content of 45 wt.-% SEBS. Film morphology analysis indicated a gradual shift in disperse phase structure and orientation, leading to phase inversion at the highest SEBS content without negatively affecting transparency.

## 1. Introduction

Polymers are the only packaging materials capable of combining transparency and flexibility, making them very useful in multiple food- and non-food flexible packaging applications, but also in the pharmaceutical sector, where specific requirements need to be met [1]. Next to avoiding potentially toxic components and limiting migration into the packaged goods, recyclability is a key aspect for many of these applications. The materials must combine flexibility to allow complete emptying of a collapsing package, toughness for safe handling and transparency for checking the content. Combining these properties while meeting legal requirements and customer expectations is, however, no trivial task.

In the medical and pharmaceutical areas especially, plasticized PVC (PVC-P) has long been a standard material, but it comes under significant pressure because of health [2,3] and environmental concerns [4,5], specifically regarding the formation of micro- and nanoplastics as a consequence of plasticizer loss [6]. Compositions based on polypropylene (PP) are, however, attracting increasing attention due to their excellent cost–performance ratio and the possibility to design copolymers in a wide property range [7]. Already, soft heterophasic copolymers (HECOs, also called impact copolymers and typically abbreviated as ICPs) based on a PP random copolymer matrix, commonly called random-heterophasic copolymers (RAHECOs), which can be produced in multi-reactor plants using modern technology [8], are capable of displaying good property combinations, but it is difficult to reach the ultimate softness for collapsible bottles or pouches while maintaining sterilizability and not exceeding the necessary limits of extractables.

**Figure 1:**
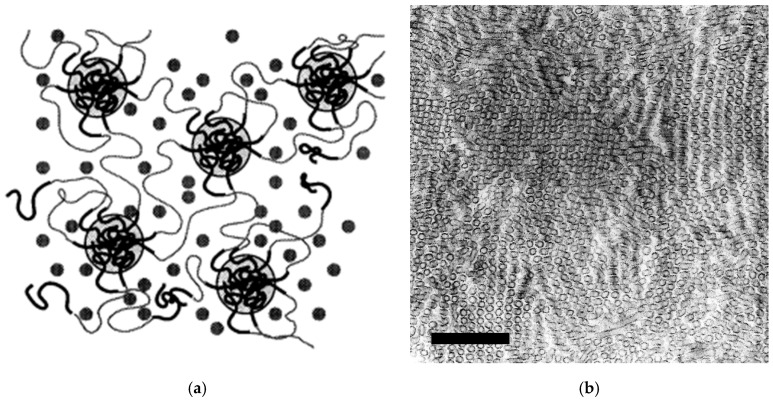
Phase morphology of an SEBS triblock copolymer: (**a**). Schematic representation (from [15], reprinted with permission from American Chemical Society); (**b**) Transmission electron microscopy image of Kraton G 1652 (styrene content 28–30 wt.-%) showing microphase-separated spherical PS domains in the amorphous EB matrix (compression-molded sample, RuO_4_ contrasting, scale bar 200 nm).

The use of external elastomers such as styrene–ethylene/butylene–styrene triblock copolymers (SEBSs) was first applied to PP random copolymers (RACOs) and later extended to more complex polyolefin systems [9,10,11,12,13,14]. SEBSs, typically applied in the form of triblock copolymers, are a class of thermoplastic elastomers without chemical cross-linking. They form a dense physical network of polystyrene (PS) spheres or rods connected covalently by elastic ethylene–butylene copolymer (EB) chains resulting from hydrogenated polyisoprene [15,16,17,18]. This morphology, which can be controlled based on synthesis, arises from microphase separation between the glassy PS end-blocks and the rubbery EB mid-block, producing well defined nanometer-scale domains whose size and geometry are governed by the relative block lengths. This morphology combines two attractive properties not easily obtained with polyolefins—optical transparency, due to the small domain size that does not scatter light in the visible wavelength, and mechanical behavior similar to a chemically cross-linked rubber due to the network structure. This two-phase structure—schematically illustrated in Figure 1—is primarily determined by the PS/EB volume ratio, with the producer-specified styrene content serving as a practical proxy for the PS block fraction. In turn, the extent of microphase separation and the resulting domain connectivity dictate the material’s flexibility, modulus, and compatibility with other polymers.

In the broad field of PP blends and compounds, SEBS has been applied primarily for impact modification [19,20,21,22,23,24,25,26] and for compatibilization in PP/PS blends [27,28,29]. The latter subject, which has not found widespread application to date despite many years of research, will not be discussed here. In contrast, impact modification by SEBS is applied on a commercial scale, not just for the here-discussed soft and flexible compositions, but also for mineral-reinforced compounds for automotive applications [30,31] and in the recycling of multi-polymer materials [32,33,34].

Only studies on PP-based systems without reinforcing fillers are relevant for the context of the present study, as transparency is a relevant target. One of the earliest systematic studies in that area with a variation of the SEBS elastomer structure comes from Stricker et al. [19], who investigated molecular weight and related particle size effects, finding that lower values of both parameters resulted in improved ductility. Concentration series with one or more types of SBS and SEBS based on single-phase PP were done by Bassani et al. [20] and Abreu et al. [21]. The latter study also shows better compatibility for a RACO in comparison to a crystalline PP homopolymer matrix, resulting in smaller dispersed particles and greater toughness. Matsuda et al. [22] compared SEBS and two further styrene elastomers in blends based on a PP homopolymer, finding better toughening effects for triblock structures and higher styrene content, but performed all tests at a constant elastomer content of 30 wt.-%.

An earlier study from our company by Grein et al. [23] used both RACOs and HECOs for modification with SEBS and/or a PE plastomer (ethylene-octene copolymer, EOC), trying to maximize impact strength in combination with transparency. The aim was to achieve a bimodal size distribution of the elastomer phase, which was only achieved for an SEBS with higher styrene content. Combining SEBS or EOC modification with α-nucleation of PP was studied by Fanegas et al. [24], again based on a homopolymer base, finding that this allowed a parallel improvement of stiffness and impact strength. More directed towards softness and transparency was the study of Ahmad et al. [25], in which high concentrations (30–70 wt.-%) of SEBS in PP were tested, and a co-continuous morphology was found at the upper limit. A similar structure was observed in the aforementioned development towards flexible medical pouches in our company [10] and by Kiehle et al. [11].

A very recent study by Sun et al. [26], which is unfortunately missing morphology information, discusses a certain retardation in crystallization of the PP matrix by elastomer addition. This could be seen as further enhancing flexibility, just like the possible addition of a low-molecular-weight paraffinic or naphthenic oil component [14], a standard approach in the case of thermoplastic vulcanizates (TPVs).

This TPV concept, i.e., the combination of styrene elastomers with cross-linking, is a possible variation possessing even higher toughness, for which different routes have been presented [35,36]. This was also tested at Borealis, using a styrene-isoprene-styrene (SIS) triblock copolymer [37]. Better toughness with cross-linking was confirmed, but the resulting materials were found to be unsuitable for film applications due to gel formation causing massive surface roughness.

The goal of this work is to compare four different PP grades, one RACO, one HECO (ICP) and two RAHECOs, regarding their ability to form soft, transparent, tough films when blended with SEBS. We combine injection-molded and cast-film testing with TEM morphology analysis to establish how matrix crystallinity and the disperse phase content (HECO, RAHECO) control the required SEBS loading. This allows identifying optimal PP base polymer and blend designs for future medical packaging formulations.

## 2. Materials and Methods

### 2.1. Components and Blends

Four different types of ethylene–propylene copolymer, all produced by Borealis GmbH (Vienna, Austria) were used as base components for the mixing series: one single-phase PP random copolymer (RACO) [38], one heterophasic or impact PP copolymer with a crystalline PP homopolymer matrix (HECO) [39], and two different RAHECO types as defined above [40,41], wherein the matrix is less crystalline than in the HECO. All four polymers are developmental materials from pilot-scale production using the multi-stage Borstar PP process and conventional or post-phthalate-type Ziegler–Natta catalyst [8], using one loop and a gas-phase reactor (GPR1) for the RACO, but loop and GPR1 for the matrix part of the HECO and RAHECO types, with one further GPR2 for the amorphous copolymer part (EPC) of HECO and RAHECO2 and, in total, 4 reactors including GPR3 for RAHECO 1, with the highest amount of EPC. Details of the production of such copolymers can be found in the literature [7] and respective patents [37,42].

All tests except electron microscopy were performed in the laboratories of Borealis in Linz according to accredited test methods, meaning that the precision levels of the respective standards will be met or exceeded in any case. The base polymers were subjected to a basic analytical characterization; Table 1 summarizes the results together with the stiffness:

- Melt flow rate (*MFR*) as a proxy for processability was determined according to ISO 1133 [43] (precision < +/− 10%) at a temperature of 230 °C and a load of 2.16 kg.
- Differential scanning calorimetry (DSC) analysis was used for determining melting temperature(s) (*T_M_*), melting enthalpy/ies (*H_M_*) and crystallization temperature (Tc). We used a TA Instrument Q200 on 5 to 7 mg samples, running DSC according to ISO 11357/part 3/method C2 [44] (*T_M_* precision < +/− 1 °C) in a heat/cool/heat cycle with a scan rate of 10 °C/min in the temperature range of −30 to +225 °C. Crystallinity *X_C_* was calculated based on a value of 170 J/g for fully crystalline PP based on the most recent extensive study of Lanyi et al. [45].
- Quantitative nuclear magnetic resonance (^13^C-NMR) spectroscopy was used to determine the ethylene (C2) content of the polymers. Quantitative ^13^C{^1^H} NMR spectra were recorded in the solution state using a Bruker Avance III 400 NMR spectrometer. All spectra were recorded using an extended temperature probe head at 125 °C using nitrogen gas for all pneumatics. Approximately 200 mg of material was dissolved in 3 mL of 1,2-tetrachloroethane-d2 (TCE-d2). The resulting NMR spectra were processed, integrated and the relevant quantitative properties determined from the integrals. All chemical shifts were indirectly referenced to the central methylene group of the ethylene block (EEE) at 30.00 ppm using the chemical shift of the solvent. The comonomer fraction was quantified using the method of Wang et al. [46] through integration of multiple signals across the whole spectral region in the ^13^C{^1H^} spectra. More details can be found in the related patents [37].
- The xylene cold-soluble (XCS) fraction was determined at 25 °C according to ISO 16152 [47] (precision < +/− 0.5 wt.-%). For the multi-phase copolymers, qualitative XCS was also used to generate a sample for determining the C2 content of said fraction by NMR and the intrinsic viscosity IV(XCS). IV was measured according to ISO 1628/1 [48] in decalin at 135 °C.

As modifier, the commercial SEBS type Kraton G 1645 of Kraton Polymers LLC, Houston, TX, USA, was applied. This thermoplastic elastomer is characterized by an MFR (230 °C/2.16 kg) of 2.0–4.5 g/10 min, a styrene content of 11.5–13.5 wt.-%, a Shore A hardness of 35 and a tensile strength of 10.3 MPa. This SEBS type has been found to be better compatible with PP copolymers [8,25] than the previously used Kraton G 1652 (styrene content 28.2–30 wt.-%) [23], resulting in higher transparency.

### 2.2. Processing and Characterization

The base polymers were mixed with varying amounts of the SEBS modifier in a co-rotating twin screw extruder, type Coperion ZSK57 of Coperion, Stuttgart, Germany, at temperatures of 200–230 °C, without adding specific extra stabilization on top of the usual combination of 0.05 wt.-% of Ca-stearate as an acid scavenger and 0.10 wt.-% of the commercial stabilizer package ‘Irganox B215 FF’, a 2:1 blend of tris-(2,4-di-t-butylphenyl) phosphite (‘Irgafos 168’ of BASF SE, Ludwigshafen, Germany) and tetrakis[methylene (3,5-di-t-butyl-4-hydroxhydrocinnamate)] methane (‘Irganox 1010’ of BASF SE). No nucleating agents were added, and strand pelletization after water-bath cooling was applied.

MFR and XCS were determined for the base polymers. The mechanical and optical performance of all blend compositions was determined first on standard injection-molded (IM) specimens, using 10 repetitions in each method:

- The flexural modulus (*FM*) was determined in a 3-point-bending test at 23 °C according to ISO 178 [49] (precision < +/− 5% relative) on 80 × 10 × 4 mm^3^ test bars injection-molded in line with EN ISO 1873-2 [50].
- The Charpy notched impact strength (*NIS*) was measured according to ISO 179 1eA [51] (precision < +/− 10% relative) at +23 or −20 °C, using the same type of specimen as for *FM*.
- Haze(IM) was determined according to ASTM D1003 [52] on plaques of 60 × 60 × 1 mm^3^ (precision < +/− 5% relative) produced by injection molding in line with EN ISO 1873-2 [50].

Subsequently, cast films were produced from the blends on a small-scale laboratory cast-film line from the company COLLIN Lab & Pilot Solutions GmbH, Maitenbeth, Germany. The line consists of three extruders (diameters A 25/C 30/B 25 mm), of which only extruder B was used, alongside a coat-hanger slit die of 300 mm width and a chill roll with an air knife, followed by a side trimmer and a winder. A melt temperature of 230 °C and a chill-roll temperature of 15 °C was used to get maximum transparency, in line with prior experience [53]. The resulting films had an as-trimmed width of 250 mm and a thickness of 50 µm.

Like the molded specimens, the cast films were stored for at least 96 h at 23 °C before testing as follows (again with 10 repetitions in each method):

- Tensile modulus (*TM*) in machine direction (*MD*) was determined according to ISO 527-3 [54] (precision < +/− 5% relative) at 23 °C at a cross-head speed of 1 mm/min.
- Haze(CF) was determined according to ASTM D1003 [52].
- A penetration test according to ISO 7765-2 [55] (precision < +/− 10% relative), also commonly known as Dynatest, was performed at 0 °C to assess film toughness, recording the total penetration energy *W*(*break*).

In addition, the morphology of the cast films was studied in detail for the blend series based on the HECO material. These investigations were performed with transmission electron microscopy (TEM) on ultrathin sections in both machine (*MD*) and transverse direction (*TD*) to assess phase structure orientation. Samples were contrasted with reactive RuO_4_ formation as an established technique for semicrystalline polymers [56,57].

## 3. Results and Discussion

**Figure 2:**
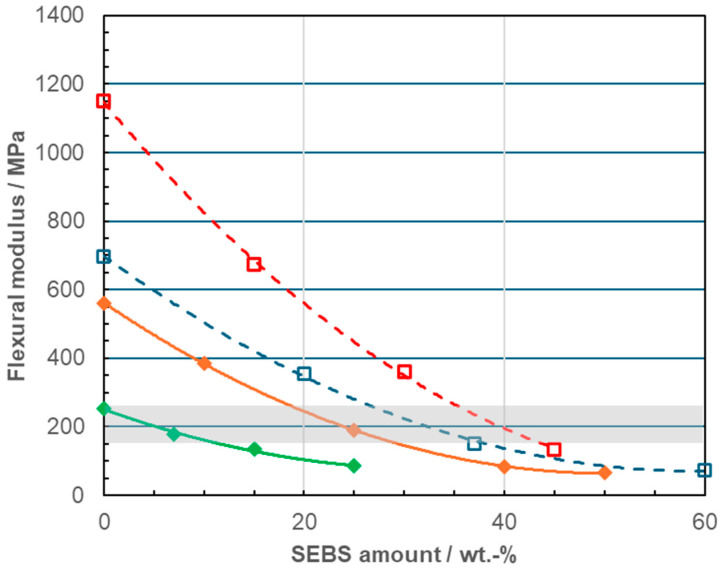
Flexural modulus of base polymers and blend compositions as a function of SEBS content; open symbols and dashed lines for RACO (blue) and HECO blends (red) and full symbols and lines for RAHECO blends (RAHECO 1 green, RAHECO 2 orange); the LDPE modulus range is indicated as a gray bar.

One of the key targets in the development of PP-based compositions for collapsible bottles or pouches is reaching the necessary flexibility, for which low-density polyethylene (LDPE) serves as a suitable reference because it is commonly used in flexible medical applications that do not require steam sterilization [58,59]. This means a flexural modulus (FM) on molded specimens of 150–200 MPa, corresponding to values given as a target in earlier studies [10,12,13]. The FM for all compositions evaluated in the present study is presented in an overview in Figure 2, showing the significant non-linearity as a function of the SEBS modifier content and the concordance of several target materials to the LDPE range.

This contrasts with the linear function found in a variation of the reactor-based EPC content by dilution with matrix polymer as done before for RAHECOs [40], meaning that no simple linear mixing rule can be applied in the present case. In previous studies with lower concentrations, this has been related to morphology effects [20,21], meaning that the base polymer morphology—single- or multi-phase, homopolymer or RACO matrix—should affect the relation.

Two further critically relevant parameters are transparency, corresponding to a low haze value, and toughness. Nucleating agents as used by Fanegas et al. [24] are largely excluded for medical applications, meaning that for the matrix crystallinity, comonomer content and quenching efficiency define transparency [37]. For the disperse phase, SEBS alone or in combination with EPC, the morphology, and the difference in refractive index are decisive for haze contribution [7]. In addition, for toughness, a critical amount of elastomer phase must be exceeded to achieve ductility [40].

### 3.1. Blends Based on Single-Phase Random Copolymer (RACO) and Impact Copolymer (HECO)

Using a RACO as base for SEBS modification was the standard approach when first moving to polyolefin-based solutions for soft medical applications [9,10]. In contrast to that, the HECO series based on a copolymer with rather C2-rich EPC to enhance the compatibility stems from the development of sterilizable food packaging like stand-up pouches, where transparency combined with limited flexibility and high toughness was required [60]. Having a rather high C2 content in the disperse phase favorably lowers the glass transition temperature as shown, for example, by Doshev et al. [61], enhancing toughness at low temperatures, but results in crystalline PE formation limiting transparency [39].

The compositions tested in these two series and their related performance are listed in Table 2. Here, the SEBS content reaches up to 45 wt.-% for the HECO (which already has a base level of ~13 wt.-% EPC, see Table 1) and up to 61 wt.-% for the initially single-phase RACO. A similar disperse phase content as expressed by the *XCS* is reached, but the resulting stiffness is clearly different, with the RACO-based blends being more flexible due to the lower crystallinity of the matrix phase (when comparing the XC values in Table 1, the XCS content must be considered).

**Figure 3:**
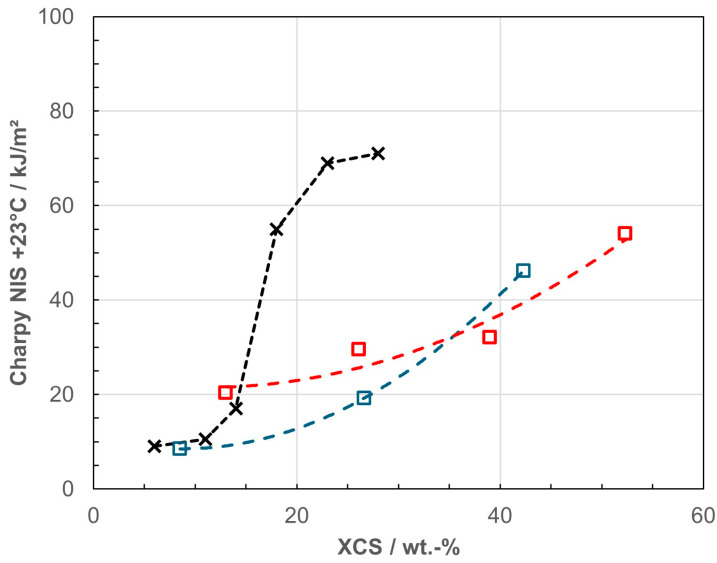
Toughness as expressed by Charpy *NIS* at 23 °C as function of *XCS* content; open symbols and dashed lines for RACO (blue) and HECO blends (red), black symbols and dotted lines for reference data from RAHECO series with variation of EPC content [40].

Toughness assessment at room temperature, as presented in Figure 3, reaches its limits for the 39/61 composition of RACO/SEBS, meaning that the last point for Charpy *NIS* at 23 °C is off the scale. Comparable data, based on the same standard and test specimen type, from a RAHECO dilution series [40], have been added to this diagram, showing that an increase in the EPC content allows reaching the brittle-to-ductile transition at lower *XCS* (even considering the 6 wt.-% of the here-used matrix); however, this involves sacrificing the flexibility presented by a blend system.

The film performance data in Table 2 show that a modulus level well below 100 MPa, i.e., exceeding LDPE and coming closer to plasticized PVC, combined with low haze and good toughness at 0 °C, can be reached in both series. This points out the key advantage of SEBS as a modifier over simply increasing EPC content.

**Figure 4:**
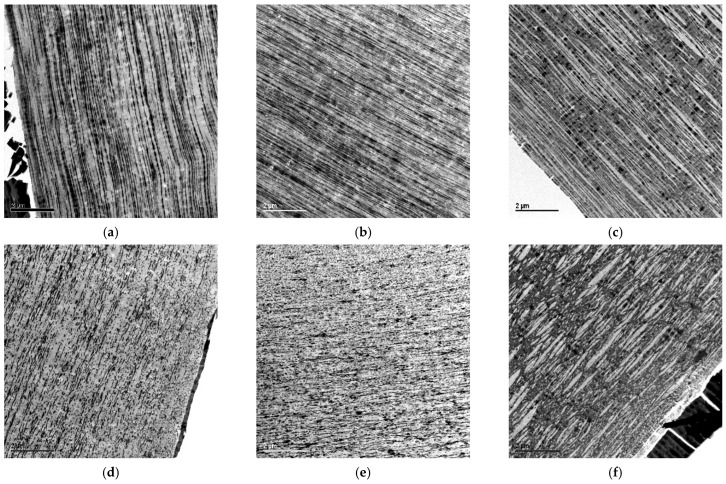
Phase morphology of cast films in MD (**top**, **a**–**c**) and TD (**bottom**, **d**–**f**) for the HECO base polymer (**a**,**d**), 85/15 blend with SEBS (**b**,**e**) and 55/45 blend with SEBS (**c**,**f**); TEM images contrasted with RuO_4_ as in [47] with 2 µm scale bars.

Details of this balance will be discussed below in relation to the RAHECO blends, but Figure 4 already presents the effect of SEBS addition to the phase morphology for the HECO series. All images were taken close to the film surface, but actual variation in the morphology across the thickness is very limited. The dispersed phase orientation is strong, as expected from the rather low IV of the EPC (see Table 1) and—in the case of modification with SEBS—a good viscosity match expressed by the very limited MFR reduction with increasing modifier content (see Table 2).

MD and TD images for the base HECO show a predominantly ‘spaghetti’-shaped disperse phase with 1-dimensional (1D) orientation, which implies a ‘pancake’ shape with 2D orientation parallel to the film surface. This is known both from similar PP/SEBS compounds [10,11], but also from automotive compositions based on PP, where the 2D-oriented particles have been shown to affect shrinkage and thermal expansion [62]. In analogy to that, films from PP/SEBS compounds sustain steam sterilization with less anisotropy in shrinkage. At the highest SEBS content of 45 wt.-%, phase inversion is observed, meaning that the crystalline PP matrix phase becomes the dispersed one (XCS is above 50 wt.-% here). This phase inversion is also described in other studies [11]; films like that are less suitable as single layers due to their inherent tackiness but can be used as the core layer in medical or other packaging systems with skin layers from other PP types [9,13].

**Figure 5:**
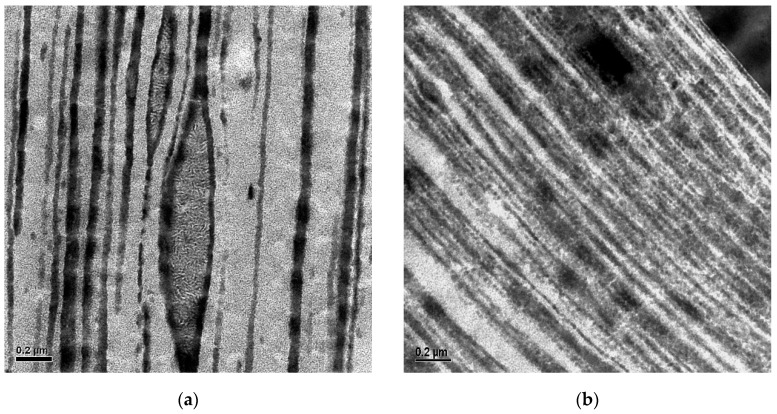
Details of the phase morphology of cast films in MD for the HECO base polymer (**a**) and the 55/45 blend with SEBS (**b**); TEM images contrasted with RuO_4_ as in [47] with 0.2 µm scale bars.

The higher resolution images in Figure 5 only show the base HECO and the phase-inverted structure of the blend with the highest SEBS content in MD. The crystalline PE core of the EPC particles is characterized by a higher lamellar thickness than the PP matrix. In film production, this core obviously resists orientation more than the amorphous part, as clearly seen for the base HECO. This clearly limits transparency, and the SEBS mixing with the amorphous EPC breaks these bigger particles into smaller and more stretched sub-units as seen for the blend. This compatibilization or ‘encapsulation’ has been observed before for this SEBS type, and no independent dispersion-generating bimodal particle size distribution occurs [23]. In this high-resolution image, the PS domains of the SEBS are also visible, similar to the structure shown in Figure 1b, but smaller due to the ~50%-lower styrene content of the applied grade.

### 3.2. Blends Based on RAHECOs

There are several motivations for moving from single-phase RACOs to RAHECOs as a blend base for soft packaging systems:

- Economically, the cost for PP copolymers is simply lower than for styrene elastomers;
- In terms of production, lowering the SEBS amount facilitates compounding and may even allow direct feeding in film production; and
- Increasing the PP content improves the possibility of recycling, which is also becoming a relevant requirement in the medical area [1,63].

Table 3 again sums up the compositions tested in the two RAHECO-based series and their related performance. Starting from a quite different level of flexibility (FM) and XCS for RAHECO 1 (254 MPa and 47.0 wt.-%) and RAHECO 2 (561 MPa and 20.5 wt.-%), the ranges of the SEBS additions are again different, reaching up to 25 and 50 wt.-%, respectively. Again, a similar disperse phase level as expressed by the XCS of ~60 wt.-% is reached, but the terminal compositions based on RAHECO 2 are softer due to a different ratio between EPC and SEBS.

**Figure 6:**
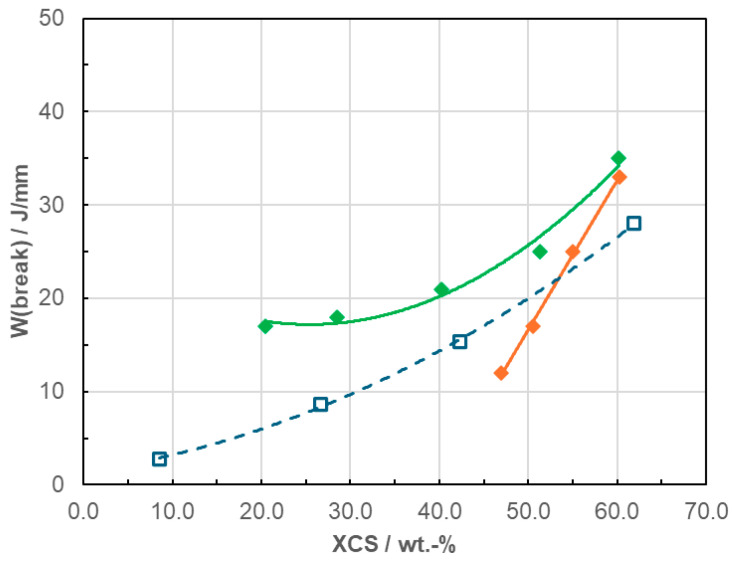
Film toughness as *W*(*break*) from penetration test (ISO 7765-2, Dynatest [54]) at 0 °C for RACO and RAHECO polymers and blend compositions as a function of SEBS content; open symbols and dashed lines for RACO (blue), full symbols and lines for RAHECO blends (RAHECO 1 green, RAHECO 2 orange).

Considering only the series targeted at soft medical applications (RACO- and RAHECO-based), the necessary flexibility can be reached in all cases, and with higher SEBS content the inherently more hazy RAHECO 2 becomes sufficiently transparent. Assessing toughness based on Charpy NIS is not possible for the whole series, as several points are outside the method range. Figure 6 therefore compares the film data of the three series, using the same reference to blend XCS as a proxy for the disperse phase like in Figure 3 above.

**Figure 7:**
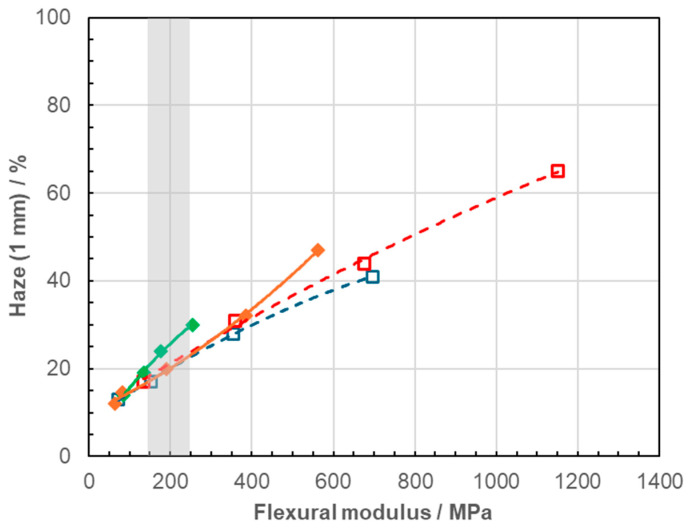
Transparency resp. Haze(IM) (ASTM D1003 [51]) for all polymers and blend compositions as a function of flexural modulus (*FM*) with increase in SEBS content from top-right to bottom-left of the diagram; open symbols and dashed lines for RACO (blue) and HECO blends (red), full symbols and lines for RAHECO blends (RAHECO 1 green, RAHECO 2 orange). The LDPE modulus range is indicated as gray bar.

The combination of EPC and SEBS has clearly improved toughness, with both RAHECO series reaching at least the onset of ductile behavior at ~60 wt.-% XCS while also achieving excellent transparency, as shown in Figure 7. Also, this speaks in favor of the advanced copolymer base for designing blends for medical applications.

## 4. Summary and Conclusions

Blends based on single- and multi-phase PP copolymers with SEBS as modifiers show attractive property combinations of flexibility, toughness and transparency as required in soft medical packaging applications like infusion pouches or blow–fill–seal bottles [1,9,10,11,12,13,59]. The replacement of plasticized PVC for such applications is already progressing rapidly in the area of medical pouches used for infusions or dialysis, and the applicability of steam sterilization also allows the substitution of LDPE.

**Figure 8:**
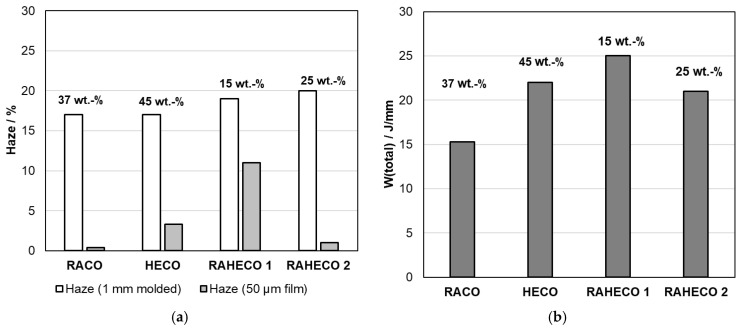
Balance of optical and mechanical properties for PP/SEBS blends with a similar flexibility of FM (molded) ~150 MPa and TM (film) ~90 MPa, percentages above the bars indicating the SEBS content; (**a**)—Haze (molded and film), (**b**)—Film toughness (Dynatest at 0 °C).

The present study compares four different base polymers using the same SEBS-type modifier, targeting the flexibility level of LDPE as reference, i.e., a flexural modulus (FM) on molded specimen in the range of 150–250 MPa. Figure 8 presents the property balance for four compositions reaching this target in terms of transparency (haze for injection-molded and or cast-film samples) and toughness (Dynatest at 0 °C on cast film). A significant reduction in the SEBS amount of 37 wt.-%, which was found necessary for single-phase RACO, is achieved for both RAHECO types. The resulting blends also show higher toughness. The HECO-based compound requires even more modifier, a sole possible advantage being the higher melting point of the matrix enabling better heat resistance. Differences in the haze relation between injection-molded plaques (1 mm thickness) and cast films (50 µm thickness) are likely related to the differences in cooling conditions [53], and for molding the use of a suitable nucleating agent (soluble-type, also called a clarifier) can improve the situation [24].

The high SEBS content of 57 wt.-% used in the substitution studies cited above for blood-bag films from plasticized PVC [12,13] is obviously not necessary for the targeted performance. In comparison to these studies, an improved level of water vapor and oxygen barrier can be expected for compositions with a higher PP content. A further argument for keeping the SEBS fraction at levels below 30 wt.-% is the increasing need for the recycling of medical articles [32,34,63].

## Figures and Tables

**Table 1:** Base polymer characteristics.

Type		RACO	HECO	RAHECO 1	RAHECO 2
***MFR* 230 °C/2.16 kg**	g/10 min	4.5	2.3	3.1	4.9
**C2 (total)**	wt.-%	4.2	6.0	11.3	9.2
***XCS***	wt.-%	8.5	13	47	20.5
**C2 (XCS)**	wt.-%	n.d.	46	19.4	33.4
***IV* (XCS)**	dl/g	n.d.	1.7	2.0	1.5
***T_M_* (DSC)**	°C	140	162	149	142
***T_C_* (DSC)**	°C	100	116	103	101
***H_M_* (DSC)**	J/g	52	80	65	62
***X_C_* (DSC)**	%	31	47	38	36
**Flexural modulus**	MPa	696	1150	254	561

**Table 2:** Composition and properties of RACO and HECO blends with SEBS.

**Base Type**		**RACO**			
**Base amount**	wt.-%	100	80	63	40
**SEBS amount**	wt.-%	0	20	37	60
***MFR* (blend)**	g/10 min	4.5	4.0	3.1	3.0
***XCS* (blend)**	wt.-%	8.5	26.6	42.3	61.9
**IM specimens**					
**Flexural modulus (*FM*)**	MPa	696	354	152	72
**Charpy *NIS* +23 °C**	kJ/m^2^	8.5	19.2	46.2	n.b.
**Haze (IM)**	%	41	28	17	13
**Cast film 50 µm**					
**Tensile modulus (*TM*)**	MPa	393	236	88	41
**Haze (CF)**	%	1.9	1.3	0.4	0.5
***W* (*break*) 0 °C**	J/mm	2.8	8.6	15.3	28.0
**Base type**		**HECO**			
**Base amount**	wt.-%	100	85	70	55
**SEBS amount**	wt.-%	0	15	30	45
***MFR* (blend)**	g/10 min	2.3	2.9	3.2	3.7
***XCS* (blend)**	wt.-%	13	26.1	39.0	52.3
**IM specimens**					
**Flexural modulus (*FM*)**	MPa	1150	675	360	135
**Charpy *NIS* +23 °C**	kJ/m^2^	20.3	29.5	32.0	54
**Haze (IM)**	%	65	44	31	17
**Cast films 50 µm**					
**Tensile modulus (*TM*)**	MPa	663	390	206	80
**Haze (CF)**	%	13.2	8.9	6.0	3.3
***W* (*break*) 0 °C**	J/mm	9.2	12.5	14.1	22.0

**Table 3:** Composition and properties of RAHECO blends with SEBS.

**Base Type**		**RAHECO 1**				
**Base amount**	wt.-%	100	93	85	75	
**SEBS amount**	wt.-%	0	7	15	25	
***MFR* (blend)**	g/10 min	3.1	4.0	4.6	4.0	
***XCS* (blend)**	wt.-%	47.0	50.5	55.0	60.3	
**IM specimens**						
**Flexural modulus (*FM*)**	MPa	254	177	135	85	
**Charpy *NIS* +23 °C**	kJ/m^2^	69	95	n.b.	n.b.	
**Haze (IM)**	%	30	24	19	14	
**Cast films 50 µm**						
**Tensile modulus (*TM*)**	MPa	198	110	82	52	
**Haze (CF)**	%	13	17	11	5	
***W* (*break*) 0 °C**	J/mm	12	17	25	33	
**Base type**		**RAHECO 2**				
**Base amount**	wt.-%	100	90	75	60	50
**SEBS amount**	wt.-%	0	10	25	40	50
***MFR* (blend)**	g/10 min	4.9	4.6	4.7	4.9	4.9
***XCS* (blend)**	wt.-%	20.5	28.5	40.3	51.3	60.2
**IM specimens**						
**Flexural modulus**	MPa	561	386	190	83	65
**Charpy NIS +23 °C**	kJ/m^2^	13.5	32	98	n.b.	n.b.
**Haze (IM)**	%	47	32	20	14.5	12
**Cast films 50 µm**						
**Tensile modulus**	MPa	322	262	101	60	35
**Haze (CF)**	%	1.0	1.0	1.0	1.0	1.0
***W* (*break*) 0 °C**	J/mm	17	18	21	25	35

## Data Availability

The majority of the data resulting from the here-presented study can be found in Table 1, Table 2 and Table 3. Further information can be obtained from the authors upon request.

## Abbreviations

The following abbreviations are used in this manuscript:

C2	Ethylene
DSC	Differential scanning calorimetry
EB	Ethylene-butene elastomer
EPC	Ethylene–propylene copolymer (elastomer)
FM	Flexural modulus
HECO	Heterophasic (impact) copolymer of PP with C2 (ICP)
IV	Intrinsic viscosity
LDPE	Low-density (high-pressure) polyethylene
MD	Machine direction (for films)
MFR	Melt flow rate (230 °C/2.16 kg)
NIS	Notched impact strength (Charpy)
PE	Polyethylene
PP	Polypropylene (isotactic)
PS	Polystyrene (atactic)
PVC	Poly(vinyl chloride)
RACO	Random copolymer of PP with C2
RAHECO	Random-heterophasic copolymer of PP with C2
SEBS	Styrene–ethylene/butylene–styrene block copolymer
SIS	Styrene-isoprene-styrene triblock copolymer
TD	Transverse direction (for films)
TEM	Transmission electron microscopy
Tm	Melting point (DSC)
XCS	Xylene cold-soluble fraction
